# Detection of Benzimidazole-Resistant *Haemonchus contortus* in Domestic and Wild Ruminants in Bosnia and Herzegovina

**DOI:** 10.3390/pathogens15010113

**Published:** 2026-01-20

**Authors:** Naida Kapo, Teufik Goletić, Adis Softić, Šejla Goletić Imamović, Srđan Gligorić, Jasmin Omeragić

**Affiliations:** 1 Veterinary Faculty, University of Sarajevo, Zmaja od Bosne 90, 71000 Sarajevo, Bosnia and Herzegovina; teufik.goletic@vfs.unsa.ba (T.G.); adis.softic@vfs.unsa.ba (A.S.); sejla.goletic@vfs.unsa.ba (Š.G.I.); jasmin.omeragic@vfs.unsa.ba (J.O.); 2 PI Veterinary Institute of the Republic of Srpska “Dr Vaso Butozan”, 78000 Banja Luka, Bosnia and Herzegovina; srdjan.gligoric@virs-vb.com

**Keywords:** anthelmintic resistance, *Haemonchus contortus*, shared pastures, livestock-wildlife interface, multi-host system, rt-qPCR

## Abstract

Gastrointestinal nematodes, particularly *Haemonchus contortus*, represent a major threat to ruminant health and productivity worldwide, largely due to the widespread emergence of anthelmintic resistance. In Bosnia and Herzegovina, benzimidazole resistance has previously been confirmed in domestic ruminants; however, data on wildlife remain lacking. Given the frequent spatial and temporal overlap between domestic and wild ruminants on shared pastures, this study aimed to investigate the occurrence of benzimidazole-resistant *H. contortus* genotypes within a multi-host system. During the 2024/2025 season, a total of 111 abomasal samples were collected from sheep (n = 20), lambs (n = 12), goats (n = 17), roe deer (n = 40) and chamois (n = 22) across four localities in Bosnia and Herzegovina (Laktaši, Banja Luka, Modriča and Višegrad). Adult *H. contortus* specimens were morphologically identified and confirmed using real-time quantitative PCR (rt-qPCR). Benzimidazole resistance was assessed by allele-specific rt-qPCR targeting the F200Y mutation in the β-tubulin isotype 1 gene. Statistically significant interspecies differences in β-tubulin genotype distribution were observed (*p* < 0.05), primarily driven by variation in the homozygous resistant (RR) genotype. High RR prevalence was detected in sheep (60%), lambs (50%) and roe deer (52.5%), whereas lower proportions were observed in chamois (27.3%) and goats (23.5%). Overall, 44.1% of all analyzed *H. contortus* isolates carried homozygous resistant alleles, indicating an advanced stage of benzimidazole resistance within this multi-host system. These findings demonstrate that benzimidazole resistance in *H. contortus* is not confined to domestic livestock but is also present in wild ruminants sharing the same grazing areas, consistent with circulation of resistant parasites within shared grazing systems.

## 1. Introduction

Gastrointestinal nematodes (GINs) remain one of the major health challenges for ruminants worldwide, with significant consequences for animal health, welfare and productivity [1]. Within the family Trichostrongylidae, the genus *Haemonchus* occupies a significant position, comprising more than ten described species. Among them, *Haemonchus contortus* and *Haemonchus placei* are the most widespread and clinically important. *Haemonchus contortus* typically parasitizes sheep and goats, while *H. placei* predominates in cattle. However, both species are capable of infecting a wide range of domestic and wild ruminants [2,3,4]. The life cycle of *H. contortus* is completed in approximately three weeks, and its blood-feeding activity in the abomasum leads to anemia, reduced productivity, and, in severe cases, host mortality [5,6,7,8]. In addition to their high pathogenicity and broad host range, *Haemonchus* species are among the nematodes most strongly affected by anthelmintic resistance. Consequently, benzimidazole resistance in *H. contortus* is now widely reported worldwide and represents a major threat to sustainable parasite control in ruminant production systems, contributing to substantial economic losses across Europe (≈€1.8 billion annually), with additional costs related to reduced treatment efficacy [9,10,11,12,13,14,15,16]. At the molecular level, benzimidazole resistance in *H. contortus* is most commonly associated with single-nucleotide polymorphisms in the isotype-1 β-tubulin gene, particularly at codons 167, 198 and 200, resulting in amino acid substitutions F167Y, E198A (and other variants at codon 198) and F200Y [17,18,19,20]. Among these, F200Y is most frequently reported as the predominant mutation under selection in field populations, whereas F167Y generally occurs at lower frequencies and codon-198 variants are detected less consistently across studies [21].

Since grazing is the predominant management system for ruminants, the shared use of pastures by domestic and wild animals has important epidemiological implications, particularly as wild species such as roe deer (*Capreolus capreolus*) and chamois (*Rupicapra rupicapra*) overlap with livestock and may serve as reservoirs or vectors for parasite transmission [22,23]. Studies have confirmed high levels of gastrointestinal parasitic infections in wild ruminants. The prevalence of *H. contortus* reached 57.6% in roe deer in Ukraine [24], exceeded 50% in Turkey [25], and its low host specificity has been demonstrated in the Alps, where it was detected in roe deer, chamois, alpine ibex and domestic ruminants [26]. These findings highlight the potential for active circulation of parasites between wild and domestic ruminant populations.

In Bosnia and Herzegovina, the first molecular evidence of anthelmintic resistance in *H. contortus* was recently confirmed. The F200Y mutation in the β-tubulin gene was detected in sheep, goats and cattle, with homozygous resistant (RR) genotypes widespread across host species (100% in goats, 77.4% in sheep and 94.7% in cattle), indicating that resistance is already well established in the country [27]. A particular risk is posed by traditional transhumance and communal grazing practices, which further facilitate the spread of resistant parasites between small and large wild and domestic ruminants [27,28]. In addition to biological and ecological factors, knowledge and practices of veterinarians and farmers play a crucial role in parasite control. Previous research on knowledge, attitudes, and practices in Bosnia and Herzegovina revealed a high level of awareness of anthelmintic resistance among veterinarians (92.45%), while farmers demonstrated a clear need for further education, especially regarding proper dosing and rational use of anthelmintics [28].

We selected *H. contortus* as the focal parasite of this study because it is considered the most pathogenic gastrointestinal nematode of ruminants, causing severe health problems, production losses and representing the most pressing global challenge with respect to anthelmintic resistance. Given that domestic and wild ruminants frequently overlap in pasture use, particularly in areas where traditional grazing practices and transhumance are common, there is a strong possibility of cross-transmission of resistant nematodes between these host populations. Therefore, the primary aim of this study was to investigate the occurrence of benzimidazole resistance in *H. contortus* isolated from both domestic and wild ruminants inhabiting the same areas.

## 2. Materials and Methods

### 2.1. Study Area

**Figure 1:**
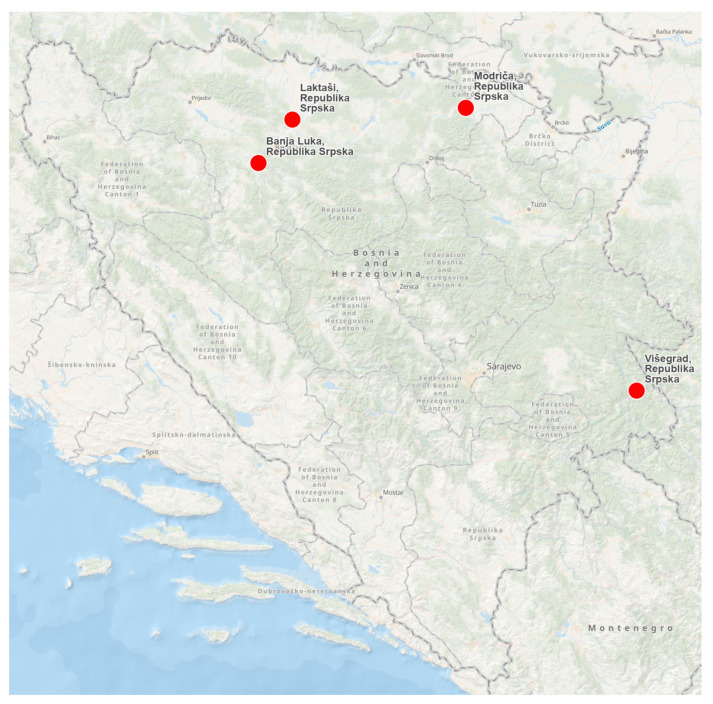
Sampling locations across Bosnia and Herzegovina, from which abomasum samples were collected from sheep, lambs, roe deer, chamois and goats. Map created using ArcGIS^®^ Online (ESRI, 641 Redlands, CA, USA).

In the 2024/2025 season, a total of 111 abomasum samples confirmed to contain adult *Haemonchus* nematodes were collected from different host species across several localities. These included 20 samples from adult sheep and 12 from lambs, 17 from goats, 40 from roe deer and 22 from chamois originating from the areas of Banja Luka, Laktaši, Višegrad and Modriča (Figure 1). Abomasum sampling was performed during hunting and slaughter of sheep, lambs, roe deer, chamois and goats under veterinary supervision. For each animal, the abomasum was removed and dissected. Following cold chain transport protocols, all samples were delivered to the laboratories of the University of Sarajevo-Veterinary Faculty.

### 2.2. Helminths

The abomasum contents (n = 111) were obtained by carefully scraping the abomasal mucosa to collect both ingested material and any parasites adhering to the mucosal surface. The collected material was then passed through a mesh or sieve to remove coarse debris and larger particles from the liquid fraction. The remaining liquid was left to stand, allowing parasites to sediment at the bottom. Portions of the sediment were subsequently examined step by step under light microscopes OLYMPUS (CH20BIMF200) and Leica (EZ4) [29,30].

### 2.3. Identification of H. contortus

Samples of *Haemonchus* were obtained from animals confirmed to be infected with *Haemonchus* parasites. Adult *H. contortus* were identified based on their morphological characteristics as described by Soulsby [31] and Lichtenfels et al. [32]. From each infected animal, one adult *H. contortus* specimen was randomly selected for further analysis (20 from sheep, 12 from lambs, 40 from roe deer, 22 from chamois and 17 from goats). Following the identification of *H. contortus* parasites, material preparation for molecular diagnostics was conducted.

### 2.4. Parasite Populations and Genomic DNA Isolation

A total of 111 adult *H. contortus* specimens were retrieved from ethanol, air-dried, and preserved at −20 °C in 1.5 mL tubes without buffer. For analysis, each worm was dissected at the level of the cervical papillae, with the uterus and eggs excluded, and the anterior portion collected for DNA extraction. Samples were homogenized for 2 min at 25 Hz using a TissueLyser II (Qiagen, Venlo, The Netherlands), and subsequently centrifuged at 14,000× *g* for 2 min. DNA was extracted using the DNeasy Blood & Tissue Kit^®^ (Qiagen, Hilden, Germany) according to the manufacturer’s instructions. The final DNA was eluted in 50 µL of nuclease-free water and stored at −20 °C until further analysis.

### 2.5. The Real-Time Quantitative PCR for Confirming H. contortus

The real-time quantitative PCR (rt-qPCR) assay for the detection of *H. contortus*, as described by Von Samson-Himmelstjerna et al. [33], was performed using genus-specific primer and species-specific probe combinations derived from the second internal transcribed spacer (ITS2) ribosomal DNA transcription unit of *H. contortus* (Table 1). The master mix consisted of 10 µL of 2× QuantiTect Probe RT-PCR Master Mix (Qiagen, Hilden, Germany), 3.5 µL of RNase-free water, 5 µL of the sample and 1.5 µL of *H. contortus*-specific primer-probe mix. The final concentration of the primers in the *H. contortus*-specific primer-probe mix was 0.7 µM, and the final concentration of the probe was 0.2 µM. RNAse-free water was used as a negative control. The cycling protocol consisted of the initial denaturation at 95 °C for 3 min, followed by 40 cycles of denaturation at 95 °C for 50 s, and annealing at 57 °C for 50 s, and extension at 72 °C for 50 s. The fluorescence was measured at the end of each annealing step. The qPCR platform used was the Magnetic Induction Cycler (MIC qPCR) from Bio Molecular Systems (Upper Coomera, Australia), and the results were analyzed using the MicPCR Software v2.2.0.

### 2.6. The Real-Time Quantitative PCR for Resistance Detection

The rt-qPCR method was employed to determine homozygous and/or heterozygous resistant genotypes based on the presence of the amino acid residue Phe at position 200 of the β-tubulin gene in *H. contortus*, using primers previously published by Humbert and Elard [34] and Arsenopoulos et al. [35].

For amplification of allele-specific products, four primers were used as follows: two allele-nonspecific primers (P1 and P4) for species detection, and two allele-specific primers for BZ resistance in *H. contortus* (P2S and P3R) (Table 2) (Eurofins Genomics Germany GmbH, Ebersberg, Germany). This approach generated three fragments, each varying in length: P1/P4 primer combination generates a fragment specific to the species (827 bp), P3R/P4 primer combination generates a fragment specific to a resistant allele (635 bp) and P1/P2S primer combination generates a fragment specific to a susceptible allele (242 bp). All four primers were pooled together in a single Primer mix, in which the concentration of P1 and P4 primers was 8 µM, and the concentration of P2S and P3R was 10 µM.

The master mix consisted of 10 µL 2 × Fast EvaGreen Master Mix (Biotium, Fremont, NH, USA), 4 µL RNase free water, 5 µL of the sample and 1 µL of a specific Primer mix in which the final concentrations of the P1 and P4 primers were 0.4 µM, and the final concentrations of the primers PS2 and P3R primers were 0.5 µM. RNase-free water was used as a negative control. The cycling protocol included an initial step of denaturation at 95 °C for 2 min, followed by 45 cycles consisting of denaturation at 94 °C for 30 s, annealing at 57 °C for 30 s, extension at 72 °C for 45 s and final extension at 72 °C for 10 min. This was immediately followed by the melting curve analysis, during which the temperature was gradually increased from 65 °C to 95 °C, with an increment of 0.3 °C per cycle. Fluorescence signals were measured and recorded at the end of each extension step, as well as continuously during the melting point analysis. The qPCR platform and the software for analysis used were the same as for rt-qPCR detection of *H. contortus.*

### 2.7. Data Management and Analysis

Each adult *H. contortus* specimen represented a single analytical unit. To avoid pseudoreplication, only one adult worm per host animal was included in the molecular analysis. Genotype frequencies were classified as homozygous resistant (RR), heterozygous resistant (RS), or homozygous susceptible (SS). Descriptive statistics were used to summarise genotype distributions across host species, and Wilson 95% confidence intervals (CI) were calculated for genotype proportions. Differences in genotype frequencies among host species were assessed using the χ^2^ test. Given the limited sample sizes in certain host groups, all inferential statistical analyses were considered exploratory. Comparisons by sampling locality were initially evaluated; however, due to uneven host distribution and small sample sizes per location, location-level stratification was not further pursued. Therefore, emphasis was placed on descriptive prevalence estimates with 95% confidence intervals and effect size patterns rather than on *p*-values alone. All statistical analyses were performed using Stata Statistical Software, version 15 (StataCorp LLC, College Station, TX, USA) [36].

## 3. Results

**Figure 2:**
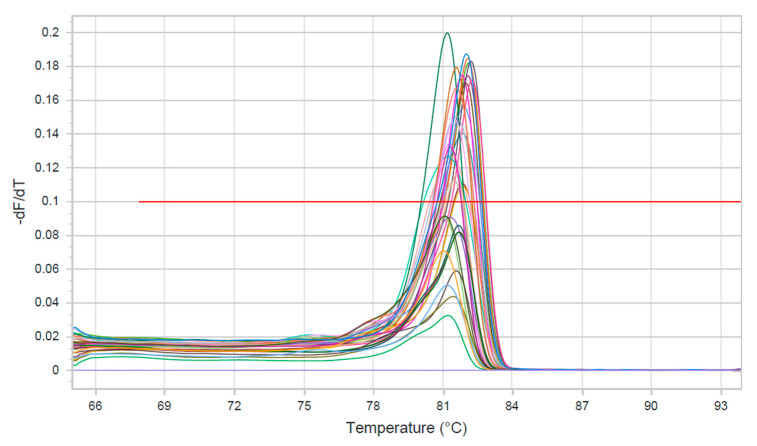
Dissociation curve analysis conducted after EvaGreen^®^ RT-qPCR amplification enabled determination of the melting temperatures (Tm) of the generated amplicons. Fluorescence signals were recorded during gradual heating from 65 °C to 95 °C. The tested samples exhibited distinct and reproducible melting peaks with Tm values between 81.2 and 82.3 °C, clearly separated from the negative control (PCR water; Tm = 70.2 °C), confirming the specificity of the amplification.

The rt-qPCR analysis confirmed that all analyzed isolates belonged to *H. contortus*. Based on allele-specific rt-qPCR targeting codon 200 of the β-tubulin isotype 1 gene, RR, heterozygous resistant (RS) and homozygous susceptible (SS) genotypes were identified across all host species (Table 3). The obtained melting temperature (Tm) and dissociation curves are presented in Figure 2. Overall, 49 out of 111 analyzed *H. contortus* isolates (44.1%) were RR, 34 isolates (30.6%) were RS, and 28 isolates (25.2%) were SS. Among domestic ruminants, RR genotypes were detected in 60% of isolates from sheep (12/20), 50% from lambs (6/12) and 23.5% from goats (4/17). In wild ruminants, RR genotypes were identified in 52.5% of isolates from roe deer (21/40) and 27.3% from chamois (6/22). The distribution of SS and RS genotypes varied numerically among host species. RS genotypes predominated in goats (47.0%) and chamois (36.3%), whereas SS genotypes were also most commonly observed in these two species (29.4% and 36.3%, respectively).

In an exploratory χ^2^ analysis, differences in genotype distribution among host species were observed (*p* < 0.05), primarily driven by variation in the RR genotype, which showed interspecies differences (*p* < 0.05). In contrast, no clear differences were detected for SS or RS genotypes among host species (*p* > 0.05). Effect size patterns indicated a higher prevalence of the RR genotype in sheep, lambs and roe deer compared with chamois and goats, while SS and RS genotypes showed only numerical variation, particularly among wildlife species.

## 4. Discussion

Benzimidazoles are among the most widely used anthelmintics in ruminant production due to their broad spectrum of activity, affordability and short withdrawal period [37,38]. However, their prolonged and often irrational use has led to reduced efficacy and the widespread emergence of anthelmintic resistance, particularly in *H. contortus* [39]. Since the 1980s, anthelmintic resistance in gastrointestinal nematodes has become a major global concern, with *H. contortus* recognized as one of the most pathogenic and economically important species owing to its haematophagous behaviour and high fecundity [40,41].

Previous studies in Bosnia and Herzegovina have documented the presence of the F200Y mutation in *H. contortus*, with RR genotypes detected in 100% of isolates from goats, 77.8% from sheep and 94.7% from cattle, while a smaller proportion of sheep (15.5%) carried RS genotypes. In contrast, SS genotypes were detected only at low frequencies in sheep (6.7%) and cattle (5.3%) [27]. Taken together, these findings clearly indicate a well-established and widespread benzimidazole resistance in domestic ruminants in Bosnia and Herzegovina.

Recent assessments of the knowledge, attitudes and practices of veterinarians and farmers in Bosnia and Herzegovina have highlighted management patterns characterized by routine anthelmintic use, approximate dosing and limited reliance on parasitological diagnostics. At the same time, traditional and extensive grazing systems, including transhumant practices, remain present in parts of the country, resulting in seasonal livestock movements and prolonged spatial and temporal overlap between domestic and wild ruminants on shared pastures [28]. Such management and production systems have important epidemiological implications, as the shared use of grazing areas facilitates the transmission and maintenance of gastrointestinal nematodes across host species. Wild ruminants such as roe deer (*Capreolus capreolus*) and chamois (*Rupicapra rupicapra*) frequently overlap spatially with livestock and may act as conduits for parasite exchange between domestic and wildlife populations [22,23]. Indeed, numerous studies have reported high prevalences of *H. contortus* in wild ruminants, including roe deer in Ukraine and Turkey, and have demonstrated low host specificity in alpine and subalpine ecosystems, where this parasite circulates among multiple wild and domestic ruminant species sharing the same grazing areas [24,25,26]. Despite these recognized risk factors, available data on benzimidazole resistance in *H. contortus* in Bosnia and Herzegovina have largely been restricted to domestic ruminants, while information on wildlife has so far been absent, particularly with regard to their potential role in maintaining, amplifying and disseminating resistant genotypes within shared grazing systems. Moreover, at the broader regional and international level, knowledge regarding the occurrence, epidemiological significance and dynamics of benzimidazole resistance in wild ruminants remains very limited [42,43].

In veterinary practice in Bosnia and Herzegovina, benzimidazoles represent one of the most frequently used anthelmintic drug classes, and their application in parasite control is therefore widespread [28]. The high proportion of resistant (R) alleles observed in small ruminants in the study area is most plausibly explained by frequent treatments and the long-term use of the same drug class without rotation, as also indicated by previous investigations. In contrast, anthelmintic treatment of wildlife is not practiced in Bosnia and Herzegovina, and hunting associations in the investigated areas have not applied anthelmintics to wild ruminants. Under these circumstances, the detection of benzimidazole-resistant *H. contortus* in roe deer and chamois is compatible with circulation of resistant parasites within shared grazing systems between domestic and wild ruminants, while the primary selection pressure is expected to occur in treated domestic livestock rather than arising from wildlife management practices.

Within this ecological framework, the present study provides clear evidence that benzimidazole resistance is not confined to domestic livestock but is also present in wild ruminants inhabiting shared pastures. Molecular analysis revealed statistically significant interspecies differences in the distribution of β-tubulin genotypes of *H. contortus*, primarily driven by variation in the prevalence of the RR genotype. High RR proportions were observed in sheep (60%) and lambs (50%), as well as in roe deer (52.5%), whereas substantially lower RR prevalences were detected in chamois (27.3%) and goats (23.5%). When all host species were considered together, nearly half of all analyzed *H. contortus* isolates (44.1%) carried RR alleles, indicating an advanced stage of benzimidazole resistance development within this multi-host system. This pattern indicates differential selection pressures among host species, likely associated with differences in exposure to anthelmintics, intensity of contact with domestic ruminants, and host–parasite ecological interactions. Of particular importance is the observation that the prevalence of the RR genotype in roe deer was similar to that recorded in domestic sheep, supporting frequent circulation of parasites among host species sharing the same pastures. In regions where traditional, extensive and transhumant grazing systems are practiced, seasonal movement of sheep flocks across lowland and upland pastures results in prolonged spatial and temporal overlap with roe deer habitats. Compared with chamois, roe deer more commonly utilize lower altitudes, open pastures, and ecotonal habitats that are regularly used during transhumant grazing by sheep and lambs, thereby increasing their exposure to parasite populations subjected to strong anthelmintic selection pressure. In contrast, the lower frequency of the RR genotype observed in chamois and goats may reflect more limited spatial and temporal interactions with intensively treated domestic herds, as well as potentially different grazing behaviour and movement patterns. Similar trends have been reported in other European studies, where wild ruminant species with more pronounced contact with domestic livestock exhibited higher proportions of resistant genotypes compared with species inhabiting predominantly mountainous or more isolated habitats. In the present study, however, the sampling localities were selected within a broadly similar production setting dominated by extensive grazing intensity, livestock density and degree of wildlife–livestock spatial overlap, and all were characterized by predominantly extensive grazing on shared pastures. Therefore, major locality-specific ecological contrasts were not expected, and the observed genotype patterns are more plausibly explained by host-related and management-related factors rather than by environmental differences between individual sites. Conversely, no statistically significant interspecific differences were detected in the prevalence of SS or RS genotypes. Although numerical variation in RS prevalence was observed, particularly in goats and chamois, these differences did not reach statistical significance. This likely reflects the transitional nature of the RS genotype, which represents an intermediate stage in the development of anthelmintic resistance and whose frequency may fluctuate depending on local selection dynamics and gene flow between parasite populations.

Evidence from other European studies supports the interpretation that resistant parasites detected in wildlife often originate from domestic hosts. In a molecular study conducted in Hungary [44], sheep similarly exhibited a high prevalence of RR genotypes (68.6%), whereas roe deer showed a considerably lower RR prevalence (17.1%), and red deer harboured exclusively susceptible genotypes. Resistant *H. contortus* detected in roe deer were therefore interpreted as resulting primarily from cross-transmission from sheep rather than from selection pressure within wildlife populations. Compared with the Hungarian findings, the substantially higher RR prevalence observed in roe deer in the present study suggests more intense or prolonged livestock–wildlife contact, likely driven by local grazing practices, spatial overlap, and the absence of targeted pasture-level measures aimed at reducing environmental contamination with infective nematode stages [28].

Comparable patterns have been reported across Europe, where resistance to benzimidazoles and macrocyclic lactones is now widespread, including in countries with relatively restrained anthelmintic use [11,12,13,14,15]. Large-scale field studies, such as those conducted in Sweden, have documented frequent treatment failure and multidrug resistance, with *H. contortus* often emerging as the dominant genus following anthelmintic treatment. A key limitation of this study is the relatively small sample size in certain host groups, particularly lambs and goats, which reduces statistical power and warrants cautious interpretation of inferential statistics. Although the data are compatible with circulation of resistant parasites between domestic and wild hosts, no population genetic, phylogenetic or multilocus analyses were performed; therefore, the directionality of transmission between domestic and wild ruminants cannot be conclusively established. Future studies integrating multilocus genotyping, whole-genome sequencing and population genetic approaches would be required to formally assess transmission pathways and directionality at the livestock–wildlife interface. To avoid pseudoreplication, molecular analysis was performed on a single adult *H. contortus* specimen per host animal. Although this approach ensures statistical independence of observations, it substantially limits inference on within-host parasite population structure and may lead to under- or overestimation of resistance allele frequencies at the host level, particularly in hosts harbouring mixed-genotype infections. Host sampling was opportunistic and depended on animal availability during routine slaughter (domestic ruminants) and hunting (wild ruminants) within each locality. Although one worm was randomly selected from each infected animal, host selection was not fully random and may have been influenced by slaughter practices and hunting pressure, potentially introducing selection bias (e.g., age, sex or body condition structure) and affecting observed genotype proportions. Detailed quantitative data on grazing intensity, livestock density and fine-scale habitat use were not collected as part of the study design, which limited more refined ecological stratification of sampling sites.

In addition, resistance genotyping was limited to the F200Y SNP in the β-tubulin isotype-1 gene. Although F200Y is widely reported as the predominant mutation under selection in field populations of *H. contortus* and represents a well-established marker for benzimidazole resistance monitoring, resistance-associated variants at codons 167 and 198 have also been described. Therefore, the prevalence of benzimidazole resistance markers reported here may be underestimated if non-F200Y variants contribute substantially to resistance in the investigated parasite populations. Inclusion of multi-SNP screening (codons 167/198/200) and analysis of multiple worms per host in future studies would provide a more comprehensive assessment of within-host diversity, improve prevalence estimates and help refine conclusions regarding resistance dynamics within multi-host grazing systems.

Beyond ecological drivers, the emergence and spread of anthelmintic resistance are strongly influenced by human decision-making. Although a relatively high level of awareness of anthelmintic resistance has been reported among veterinarians in Bosnia and Herzegovina, gaps remain in the consistent application of evidence-based parasite control strategies at the farm level. Routine anthelmintic administration, approximate dosing and the use of treatments without prior parasitological diagnosis may contribute to sustained selection pressure and accelerate resistance development.

Taken together, the findings of this study are compatible with a scenario in which resistant parasite populations circulate between domestic and wild ruminants through shared grazing systems, superimposed on strong selection pressure arising from long-term anthelmintic use in livestock. Under such conditions, wild ruminants may act as recipients and secondary disseminators of resistant parasites, or alternatively as partial refugia for susceptible genotypes, depending on local ecological connectivity and management practices. However, given the limitations of single-locus genotyping and the absence of population-level genetic analyses, the relative contribution of wildlife to the maintenance and spread of resistant genotypes cannot be conclusively determined.

Effective management of anthelmintic resistance therefore cannot rely solely on pharmacological approaches. An integrated approach that links livestock management, wildlife ecology and evidence-based decision-making is needed. Incorporating wildlife into resistance surveillance, improving access to affordable diagnostics and strengthening education on targeted selective treatment are key steps toward preserving the efficacy of existing anthelmintics and ensuring sustainable parasite control in Bosnia and Herzegovina.

## Figures and Tables

**Table 1:** Primer and probe sequences for the detection of *Haemonchus contortus* [33].

Primer-Probe	Sequence
Hc 2 multi 307T	5′-FAM-TGGCGACGATGTTC-MGB-3′
Hc 2 multi 272F	5′-GCGAATATTGAGATTGACTTAGATAGAGAC-3′
Hc 2 multi 349R	5′-GCTCAGGTTGCATTATACAAATGATAAA-3′

**Table 2:** Primers used for rt-qPCR for targeting the position 200 of the β-tubulin protein of the *Haemonchus contortus* helminths [34,35].

Primer	Sequence	Source
P1	Fw: 5′-GTCCCACGTGCTGTTCTTGT-3′	[35]
P2S	Rv: 5′-TACAGAGCTTCATTATCGATGCAGA-3′	[35]
P3R	Fw: 5′-TTGGTAGAAAACACCGATGAAACATA-3′	[35]
P4	Rv: 5′-GATCAGCATTCAGCTGTCCA-3′	[35]

**Table 3:** Distribution of homozygous resistant (RR), heterozygous resistant (RS), and susceptible (SS) genotypes of *H. contortus* identified in isolates from sheep, lambs, roe deer, chamois and goats in Bosnia and Herzegovina.

Species	SS	95% CI	RS	95% CI	RR	95% CI
Sheep	4/20 (20%)	8.1–41.6	4/20 (20%)	8.1–41.6	12/20 (60%)	38.7–78.1
Lambs	3/12 (25%)	8.9–53.2	3/12 (25%)	8.9–53.2	6/12 (50%)	25.4–74.6
Roe deer	8/40 (20%)	10.5–34.8	11/40 (27.5%)	16.1–42.8	21/40 (52.5%)	37.5–67.1
Chamois	8/22 (36.3%)	19.7–57.0	8/22 (36.3%)	19.7–57.0	6/22 (27.3%)	13.2–48.2
Goats	5/17 (29.4%)	13.2–53.1	8/17 (47%)	26.2–69.0	4/17 (23.5%)	9.6–47.3
Total	28/111 (25.2%)	18.1–34.0	34/111 (30.6%)	22.8–39.7	49/111 (44,1%)	35.3–53.4

## Data Availability

All relevant data are included in this article.
